# Coverage of Two-Dose Preemptive Cholera Mass Vaccination Campaign in High-Priority Hotspots in Shashemene, Oromia Region, Ethiopia

**DOI:** 10.1093/cid/ciae233

**Published:** 2024-07-12

**Authors:** Se Eun Park, Abel Gedefaw, Dejene Hailu, Yeonji Jeon, Ondari D Mogeni, Geun Hyeog Jang, David Mukasa, Ramzi Mraidi, Deok Ryun Kim, Tomas Getahun, Edlawit Mesfin Getachew, Biruk Yeshitela, Samuyel Ayele Abebe, Mukemil Hussen, Yeshambel Worku Demlie, Mekonnen Teferi

**Affiliations:** Clinical, Assessment, Regulatory, Evaluation (CARE) Unit, International Vaccine Institute, Seoul, Republic of Korea; Department of Global Health and Disease Control, Yonsei University Graduate School of Public Health, Seoul, Republic of Korea; Clinical, Assessment, Regulatory, Evaluation (CARE) Unit, International Vaccine Institute, Seoul, Republic of Korea; College of Medicine and Health Sciences, Hawassa University, Hawassa, Ethiopia; Clinical, Assessment, Regulatory, Evaluation (CARE) Unit, International Vaccine Institute, Seoul, Republic of Korea; School of Public Health, College of Medicine and Health Sciences, Hawassa University, Hawassa, Ethiopia; Clinical, Assessment, Regulatory, Evaluation (CARE) Unit, International Vaccine Institute, Seoul, Republic of Korea; Clinical, Assessment, Regulatory, Evaluation (CARE) Unit, International Vaccine Institute, Seoul, Republic of Korea; Biostatistics and Data Management (BDM) Department, International Vaccine Institute, Seoul, Republic of Korea; Biostatistics and Data Management (BDM) Department, International Vaccine Institute, Seoul, Republic of Korea; Biostatistics and Data Management (BDM) Department, International Vaccine Institute, Seoul, Republic of Korea; Biostatistics and Data Management (BDM) Department, International Vaccine Institute, Seoul, Republic of Korea; Clinical Trials Directorate, Armauer Hansen Research Institute, Addis Ababa, Ethiopia; Clinical Trials Directorate, Armauer Hansen Research Institute, Addis Ababa, Ethiopia; Bacterial and Viral Disease Research Directorate, Armauer Hansen Research Institute, Addis Ababa, Ethiopia; Data Science Division, Armauer Hansen Research Institute, Addis Ababa, Ethiopia; Diseases Surveillance and Response Directorate, Ethiopia Public Health Institute, Addis Ababa, Ethiopia; Diseases Surveillance and Response Directorate, Ethiopia Public Health Institute, Addis Ababa, Ethiopia; Clinical Trials Directorate, Armauer Hansen Research Institute, Addis Ababa, Ethiopia

**Keywords:** Preemptive mass vaccination campaign, Cholera, oral cholera vaccine (OCV), OCV coverage, Ethiopia

## Abstract

**Background:**

Cholera is a public health priority in Ethiopia. The Ethiopian National Cholera Plan elaborates a multi-year scheme of oral cholera vaccine (OCV) use. Aligned with this, a preemptive OCV campaign was conducted under our Ethiopia Cholera Control and Prevention project. Here, we present the OCV vaccination outcomes.

**Method:**

Cholera high-priority hotspots in the Oromia Region, Shashemene Town (ST) and Shashemene Woreda (SW), were selected. Four kebelles (Abosto, Alelu, Arada, and Awasho) in ST and 4 clusters (Faji Gole, Harabate, Toga, and Chabi) in SW were study sites with OCV areas nested within. A total of 40 000 and 60 000 people in ST and SW, respectively, were targeted for a 2-dose OCV (Euvichol-Plus) campaign in 11–15 May (first round [R1]) and 27–31 May (second round [R2]) 2022. Daily administrative OCV coverage and a coverage survey in 277 randomly selected households were conducted.

**Results:**

The administrative OCV coverage was high: 102.0% for R1 and 100.5% for R2 in ST and 99.1% (R1) and 100.0% (R1) in SW. The coverage survey showed 78.0% (95% confidence interval [CI]: 73.1–82.9) of household members with 2-dose OCV and 16.8% (95% CI: 12.4–21.3) with no OCV in ST; and 83.1% (95% CI: 79.6–86.5) with 2-dose OCV and 11.8% (95% CI: 8.8–14.8) with no OCV in SW. The 2-dose coverages in 1–4-, 5–14-, and ≥15-year age groups were 88.3% (95% CI: 70.6–96.1), 88.9% (95% CI: 82.1–95.7), and 71.3% (95% CI: 64.2–78.3), respectively, in ST and 78.2% (95% CI: 68.8–87.7), 91.0% (95% CI: 86.6–95.3), and 78.7% (95% CI: 73.2–84.1) in SW.

**Conclusions:**

High 2-dose OCV coverage was achieved. Cholera surveillance is needed to assess the vaccine impact and effectiveness.

## INTRODUCTION

Cholera is a public health priority in Ethiopia, where the disease is endemic, and epidemics occur frequently. In the past 5 years, several cholera outbreaks were declared by the Ethiopian government. A total of 215 205 cholera/acute watery diarrhea cases, 2355 deaths with a cumulative case fatality rate (CFR) of 1.1% (95% confidence interval [CI]: 1.092–1.095), and a mean annual incidence rate of 8.9 (95% CI: 6.5–11.3) per 100 000 population were reported in the last 2 decades (from January 2001 to November 2023) [1]. Since 2015 until November 2023, 99 945 cases and 1030 deaths with a cumulative CFR of 1.03% (95% CI: 1.02–1.04) have been reported [1]. In the ongoing 2023 outbreak alone, 27 101 cases and 370 deaths are reported with a case fatality rate of 1.4% and an attack rate of 24.4 per 100 000 [1]. Under-reporting of cases are possible for populations living in remote or conflict areas with poor access to healthcare facilities; not necessarily representing the true picture of disease burden [2].

In response to the cholera outbreaks from 2019 to 2023, a total of about 19 113 386 doses of oral cholera vaccine (OCV) were delivered from the global OCV stockpile; approximately 90.4% of the approved doses (21 148 800) by the International Coordinating Group, 59.9% of the requested doses (31 899 576) by the Ethiopian government [3]. In parallel, the Ethiopian government has demonstrated its commitment in tackling the country's long-standing cholera issue by developing and launching the National Cholera Elimination Plan (NCP) in 2022 [4]. The Ethiopian NCP embraced a multi-sectoral and multi-year approach, following the World Health Organization (WHO) Global Task Force on Cholera Control guidelines [5], including plans on the use of OCV, surveillance, case management, water, sanitation, and hygiene, and community engagement.

The NCP elaborates the use of OCV through reactive vaccination in outbreaks and preemptive vaccinations in cholera hotspots [4], in conjunction with other cholera prevention and control strategies. The administration of preemptive OCV planned in cholera hotspots mapped in the NCP elaborated all people ≥1 year old as eligible target population, including special population groups (pregnant women, lactating mothers, and HIV-infected persons) [4], as recommended by the 2017 WHO OCV position paper [6]. The NCP outlined the preemptive OCV vaccination campaign to target 98% of population of all 118 cholera hotspot woredas in Ethiopia in the first 5 years of the plan, followed by phased vaccination every year [4]. The plan included 2-dose OCV administration to maximize immunity, with a minimal 2-week dose interval.

This translated to a target population of about 15.5 million, with nearly 31 million doses of OCV required from 2021 until 2025, over or nearly 6 million doses annually for preemptive vaccination campaigns [4]. However, a shortage in global supplies of OCV doses has led to a temporary suspension of the 2-dose vaccination strategy and preemptive campaigns, and priority has been given to reactive vaccinations for immediate cholera epidemic controls with a single-dose strategy, announced by the International Coordinating Group (composed of members of the WHO, the International Federation of Red Cross, Médecins Sans Frontières, and the United Nations Children’s Fund (UNICEF)) on 19 October 2022 [7]. This will inevitably delay the rollout of the planned preemptive vaccination strategies in Ethiopia.

Licensed cholera vaccines include Dukoral, Shanchol, and Euvichol/Euvichol-Plus, available in the market [8, 9], and mORCVAX, licensed and produced in Vietnam [10]. Of these, the WHO prequalified Shanchol and Euvichol/Euvichol-Plus have been exclusively supplied to the global stockpile. As Shanchol will no longer be produced and supplied for the global stockpile, Euvichol-Plus remains the only OCV that continues to be supplied for global health usage through the stockpile mechanism, until new suppliers come on board [11]. Studies demonstrated the safety and efficacy (66%–85%) of 2 doses of OCV, with inferred herd immunity lasting up to 5 years in the case of Shanchol [10]. A noninferiority trial comparing Euvichol with Shanchol in the Philippines showed 2 doses of Euvichol to be noninferior to 2 doses of Shanchol in adults (82% vs 76%, respectively) and children (87% vs 89%) [12].

Findings of a matched case-control study conducted in 2012 in Guinea suggested that vaccination with 2 complete doses of Shanchol was associated with significant protection against cholera (effectiveness, 86.6%) [13]. A test-negative case-control study conducted in Odisha, India, after an OCV mass vaccination campaign in 2011 using Shanchol [14], resulted in adjusted protective effectiveness (69%) in persons receiving 2 doses, while a single dose provided a lower protection (33%) [15]. However, findings of a case-cohort study in Juba, South Sudan, conducted in 2015 after single-dose Shanchol OCV vaccination in an outbreak setting, suggested short-term protection of 80.2%–87.3% effectiveness [16]. OCV efficacy and effectiveness studies generate important information on the vaccine performance and impact in real-world populations in different settings [17]. More studies have been published on the effectiveness of Shanchol [18–21], but only a few on Euvichol-Plus; a case-control study after a mass vaccination campaign in response to a cholera outbreak in 2017–2018 in Lusaka, Zambia, found 81.0% effectiveness with 2 doses of Euvichol-Plus [22].

Shashemene Town (ST) and Shashemene Woreda (SW), located in the West Arsi zone in the Oromia region of Ethiopia, were among 104 woredas of high-priority cholera hotspots in the Ethiopian NCP [4]. Some parts of SW have a history of Shanchol OCV vaccination in 2015, which was the first mass OCV vaccination campaign in a cholera-endemic setting in Africa and outside Asia [23]. A bridging trial of Shanchol OCV was conducted to demonstrate safety and immunogenicity in the local population before this mass vaccination [19]. This introduction of Shanchol OCV in Ethiopia targeted about 62 161 people in subareas of SW, demonstrating the feasibility of mass cholera vaccination through the existing health system at affordable cost and acceptability of OCV in the community with 65% 2-dose coverage [23]. However, more than 7 years after the 2015 vaccination, about 573 000 people living in Shashemene districts (292 000 in ST and 281 000 in SW) remain exposed to cholera infection and transmission according to the cholera hotspot mapping in NCP.

To prevent cholera outbreaks, a preemptive OCV vaccination was planned as part of the Ethiopia Cholera Control and Prevention (ECCP) project, rolled out in collaboration between the International Vaccine Institute (IVI) and Armauer Hansen Research Institute (AHRI) within the Ministry of Health in Ethiopia. This 2-dose preemptive OCV vaccination campaign had been planned in 2020 and conducted in May 2022, before the single-dose OCV strategy announcement by the International Coordinating Group in October 2022. The vaccination campaign targeted approximately 100 000 people living in both urban and rural areas of Shashemene. Furthermore, sentinel healthcare facility-based cholera and diarrheal disease surveillance has been set-up and strengthened to investigate the impact and effectiveness of 2-dose Euvichol-Plus OCV vaccination in ST and SW, which will be analyzed and presented separately as the surveillance is ongoing. Here, we present the coverage of the OCV mass vaccination campaign conducted, which is essential and recommended by the WHO Global Task Force on Cholera Control to ensure high-quality provision of vaccination and the development of future recommendations for OCV use [24].

## METHODS

### Vaccination Site and Population

Shashemene districts were shortlisted as potential study areas based on the cholera hotspots mapped in Ethiopia NCP [4], and final site selection on the specific target areas for OCV vaccination was made in early 2021 based on a set of site selection criteria (Table 1). Cholera high-priority hotspots were screened, and areas with previous exposure to OCV in the last 3–5 years were excluded or less prioritized. The Ethiopian government's multi-year OCV roadmap in NCP was reviewed to ensure harmonization between various OCV vaccination plans and target areas across the country. Areas with any complementary cholera prevention measures or limited water, sanitation, and hygiene were also considered, as well as feasibility of study implementation, such as security and safety, accessibility to site, nonmobile population, proximity to existing functioning laboratories, and willingness to collaborate.

**Table 1.** Site Selection Criteria for OCV Vaccination

Step 1: Study site selection
Cholera hotspots
Cholera case numbers (crude case numbers) Cholera incidence (crude incidence per 100 000 or per 1000) Persistent cases (cholera cases reported persistently during the last 3–5 consecutive years)
Previous exposure to OCV (exclusion criteria)
Areas/population with previous exposure to OCV in the last 3–5 years were excluded or less prioritized
Plan for future OCV introduction and other complementary cholera prevention measures
Multi-year OCV introduction roadmap in NCP was considered to ensure harmonization between various OCV vaccination plans and target areas in the next years Other cholera-preventive measures, such as WaSH intervention, were considered to ensure a comprehensive approach but also to target cholera hotspots with limited WaSH, facing a higher risk of cholera outbreaks
Feasibility
Security and safety context Accessibility to site Non-mobile population Proximity to existing functioning laboratories
Step 2: Subarea selection assumption and algorithm for vaccination
Assumption [25]
In cholera-endemic areas: 30% OCV coverage would result in a 76% (95% CI: 44–95) reduction in cholera incidence for population area covered 50% OCV coverage would result in 89% (95% CI: 72–98) reduction in cholera cases among unvaccinated and 93% (95% CI: 82–99) reduction overall in the entire population
Algorithm to predefine vaccination target population and subareas within sites selected
Estimate 30–50% of the total number of populations living in sites selected (in step 1) Identify subareas (kebelles) within the sites selected that meet the population size of 30–50% of the total population in those selected sites Clearly define vaccination target population and subareas with demarcation of boundaries of selected subareas

Abbreviations: CI, confidence interval; NCP, National Cholera Plan; OCV, oral cholera vaccine; WaSH, water, sanitation, and hygiene.

Within the ST and SW, a network of sentinel healthcare facilities was established for a prospective cholera and diarrheal disease surveillance. Four kebelles (Abosto, Alelu, Arada, and Awasho) in ST and 4 clusters (Faji Gole, Harabate, Toga, and Chabi) in SW were selected as the ECCP surveillance catchment area (Table 2). The 4 clusters in SW included 22 kebelles. The surveillance catchment population was around 163 546 in ST and 162 212 in SW, of the entire population of around 291 589 in ST and 281 247 in SW. The OCV vaccination target areas were nested within the surveillance catchment area. As the total number of people planned for vaccination was approximately 40 000 (82 000 doses with 2.5% buffer for 2-dose vaccination) in ST and 60 000 (120 360 doses with 0.3% buffer for 2-dose vaccination) in SW, subareas (kebelles) within the surveillance catchment area were chosen based on an assumption and algorithm for vaccination (Table 1).

**Table 2.** ECCP Surveillance Catchment Area and OCV Target Area Populations

Study Area (Total Population No.)	Study Area Kebelles	Total Population No. for Surveillance Catchment Area	Population No. for OCV Target Area	Proportion of Surveillance Catchment Population Targeted for OCV Vaccination, %
ST (291 589)^a^	All study area kebelles	163 546	50 690	31
	Abosto	51 857	16 069	31
	Alelu	34 365	10 651	31
	Arada	37 391	11 591	31
	Awasho	39 933	12 379	31
SW (281 247)^b^	All study area clusters/kebelles	162 212	48 460	30
	Faji Gole cluster	44 232	15 852	36
	Alleli Illuu	8299	8032	97
	Bute Filichaa	8080	7820	97
	Chefa Gutaa	4624	0	n.a
	Faji Golee	9571	0	n.a
	Filich Goba	8521	0	n.a
	Kubii Gutaa	5137	0	n.a
	Harbate cluster	46 850	19 677	42
	Abaaroo	11 144	0	n.a
	A/Harabaatee	9509	0	n.a
	Awaashoo	9980	9658	97
	Ebichaa	5865	0	n.a
	E/Burqaa	10 352	10 019	97
	Toga cluster	26 304	6188	24
	Bulchana Dannabaa	6394	6188	97
	D/Calalaqa	4191	0	n.a
	M/Dammaa	7171	0	n.a
	Q/Borojjoota	5224	0	n.a
	Togaa	3324	0	n.a
	Chabi cluster	44 826	6743	15
	Bura Borema	6967	6743	97
	Chabi Dida Gnata	7018	0	n.a
	Chulule Habera	4943	0	n.a
	Kore Rogicha	6333	0	n.a
	Oine Chefo Umbure	12 060	0	n.a
	Tatesa Dedesa	7505	0	n.a

Abbreviations: n.a, not applicable; OCV, oral cholera vaccine; ST, Shashemene Town; SW, Shashemene Woreda.

^a^ST has total of 8 kebeles (Abosto, Alelu, Arada, Awasho, Bulchana, Burka Gudina, Dida Boke, and Kuyera) with a total population size of 291 589. Of the total, the 4 kebeles listed in this table were selected as the study surveillance catchment area, and the OCV target area was nested within the catchment area.

^b^SW has total of 36 kebeles (F/Goba, B/Filichaa, C/Gutta, Alleeli Illuu, A/Harabaatee, E/Burqaa, Awaashoo, Abaaroo, Ebichaa, Q/Borojjoota, B/Dannabaa, M/Dammaa, Togaa, D/Calalaqa, Chabi Dida Gnata, X/Daddeesa, Bu/Boramaa, Korea/Roogicha, C/Habaara, O/C/Umbure, J/Qorkee, T/W/Elemo, H/Qundhii, Karara Filichaa, Mararroo, I/Qorkee, J/Diidaa, O/Jalloo, Wshagulee, A/Shiifa, F/Solee, Dannisaa, Gonde Qarsoo, J/Wondaree, H/Siimboo, F/Solee) with a total population size of 281 247. Of the total, the 22 kebeles listed in this table were selected as the study surveillance catchment area, and the OCV target area was nested within the catchment area.

The proportion of the vaccination target population was formulated considering the Euvichol-Plus OCV effectiveness research scope embedded in the project, and the budget ceiling also limited the number of doses for procurement. Vaccinating the entire populations in the surveillance catchment area would result in reducing cholera cases after vaccination, considering the impact and efficacy of the OCV, making it difficult to conduct effectiveness research (i.e., hard to reach minimum sample size) though desirable for public health goal (i.e., the more people are vaccinated, the better). In our attempt to balance research needs and public health goals, an existing modeling on OCV herd protection was referenced. A large-scale stochastic cholera transmission model for Matlab, Bangladesh, estimated that in cholera-endemic areas a modest 30% OCV coverage would result in a 76% (95% CI: 44–95) reduction in cholera incidence for the population area covered [25]. Based on this assumption, about 30% of the surveillance catchment area populations living in ST and SW were selected (Table 1). Vaccination target populations and kebelles were clearly defined before study rollout. The surveillance catchment areas included kebelles targeted with and without OCV vaccinations in ST and SW, enabling the areas with similar characteristics as a background setting for a case-control vaccine effectiveness study after the preemptive 2-dose Euvichol-Plus OCV vaccination campaigns.

### Vaccination Strategy and Microplanning

A total of about 202 360 doses of Euvichol-Plus OCV were procured and delivered to Addis Ababa entry port in Ethiopia under the cold chain (2–7°C). The OCV doses were transported to a central vaccine storage facility of the Ethiopia Pharmaceuticals Supplies Agency (EPSA) in Addis Ababa for in-country handling, management, and storage. Before the vaccination dates, the vaccine doses were delivered to the vaccine storage facilities and healthcare facilities in ST and SW. Throughout the entire process, the vaccine cold chain was managed with daily temperature monitoring of vaccine storage conditions, and vaccine delivery and cold chain logs were documented. Microplanning of the OCV mass vaccination campaign included engaging local government officials, health professionals, field workers, community leaders, health extension workers (HEWs), and study team members. A mixed vaccination strategy that included both fixed posts and mobile teams was adapted to ensure that those residing more remotely within the vaccination target areas were reached. Trained health professionals and field workers conducted community sensitization on the OCV vaccination campaign before and during each round of vaccinations. OCV vaccination cards, vaccination registries, and other materials in the microplan (Supplementary Materials) and vaccine cool boxes were prepared and prepositioned at each vaccination post and with vaccination teams.

### Vaccination Coverage Estimation

OCV vaccination coverage was assessed in 2 ways: administrative coverage was monitored daily, and a vaccine coverage survey was conducted at the end of the vaccination campaign. Administrative coverage was monitored at the end of each day during both rounds of vaccination. The daily vaccine coverage rate (VCR) was accumulated to evaluate the administrative VCR for each round of vaccination. The administrative VCR was also assessed in an age group–stratified analysis (comparing ages 1 to <5, 5 to <15, and ≥15 years). Daily monitoring of the administrative VCR also helped the vaccination teams to reach more people for vaccination and increase coverage in subsequent days.

The vaccine coverage survey was performed through household questionnaires in the vaccination target areas. Sample size was calculated using a statistical formula. Based on a 2-sided 95% CI, accounting for a design effect of 2, a dropout rate of 10%, vaccination coverage of approximately 70%, and reasonable precision set at 10%, sample size requirements were determined following the formula recommended by the WHO [26]. A minimum sample size of about 273 households was required to estimate vaccine coverage in the vaccination areas, considering the population structure with age group–stratified minimum sampling. After microplanning, total 277 households were proportionally allocated to ST and SW based on the total number of populations vaccinated: 112 and 165 households in ST and SW, respectively.

For the OCV coverage survey, households were randomly selected from areas where the vaccination campaign was conducted using the following approach in ST and SW. In ST, the list of households and population in each kebele were obtained from the ST Health Directorate. Households were randomly selected from each kebelle/ketena (lowest local government administrative units in Ethiopia) targeted for the OCV vaccination campaign. The minimum number of households to be sampled was determined by considering the total number of households vaccinated in each kebelle/ketena (Supplementary Table 1). The vaccine coverage survey team was able to access the selected households with the help of HEWs assigned by the ST Health Directorate. In SW, since a complete list of households in each kebelles was not available, the households were randomly selected from each kebelle within 4 clusters where 2 doses of OCV were administered (Supplementary Table 2). Households were proportionally allocated to the kebelles, considering the total number of households in each kebelle selected for OCV administration.

Each OCV coverage survey team was composed of an HEW, a research assistant, and a supervisor. Once a household was identified, written informed consent was obtained, and a survey questionnaire was asked and data collected electronically using tablets (REDCap system). The status of OCV vaccination was verified through OCV vaccination cards given to the individuals, when OCV doses were administered during the vaccination campaign (Supplementary Material 1). If the interviewed household heads and/or family members had lost or could not find the OCV vaccination cards for verification, the status of OCV vaccination was recorded based on the verbal confirmation.

### Statistical Analysis

Categorical variables were summarized as frequencies and percentages. The standard error (SE) for the OCV coverage percentages was calculated considering the design effect, to account for the clustering within the sampling strategy. The SE was approximated using the formula


$$\mathrm{SE}(P)=\sqrt{\mathrm{DEFF}\;.\;\frac{P.(100-P)}{n}},$$

where *P* is the percentage, *n* is the total sample size for each subgroup, and DEFF is the design effect for the survey [27]. The CIs then informed the precision and reliability of coverage estimates. All statistical analyses were performed using SAS software, version 9.4 (SAS Inc., Cary, NC, USA).

### Ethics Approval and Public Involvement

The study protocol, including the OCV vaccination and coverage survey, has obtained research ethics approval by the IVI Institutional Review Board (IRB), Seoul, Korea (IRB no. 2021-005); the AHRI/ALERT Ethics Review Committee, Addis Ababa, Ethiopia (approval letter dated 28 July 2021; form AF-10-015); Ethiopian National Research Ethics Review Committee, Addis Ababa, Ethiopia (approval letter dated 17 December 2021; reference no. 7/2-512/m259/35); Oromia Region Health Bureau (Oromia Health Research Directorate approval letter dated 26 August 2021; reference no. REF/UBTU/516/10239).

## RESULTS

### OCV Vaccination Administrative Coverage by Kebelle and Age Group in Planned Vaccination Areas

The overall administrative coverage of OCV vaccination campaign in the planned vaccination areas was high. In ST, 41 056 (102.0%) and 40 453 (100.5%) people received the OCV during the first round (R1) and second round (R2) of the vaccination campaign, respectively, more than the planned 40 250 people (Table 3). Among the vaccinated populations in ST, 270 people received the first dose during R2, and 40 183 received the complete 2 doses of OCV. In SW, 60 502 (99.1% of 61 039 planned) people received the OCV during R1 and 60 480 (100.0% of 60 502 planned) during R2. Among those vaccinated in SW, 344 people received the first dose during R2, and 60 136 people received the complete 2 doses. The OCV administrative coverage by kebelle was equally high (99.0–115.8), with slight variations: 97.1% coverage during R2 in Abosto in ST and 87% in Abaro, 90.8% in Bura Borama, and 92.8% in Faji Gole during R1 in SW (Table 4).

**Table 3.** OCV Vaccination Administrative Coverage in Shashemene Town and Shashemene Woreda

Study Area and Vaccination Round	Vaccination Population, No.		Administrative Coverage, %
	Planned	Actual	
ST			
R1	40 250	41 056	102.0
R2	40 250	40 453^a^	100.5
SW			
R1	61 039	60 502	99.1
R2	60 502	60 480^b^	100.0

Abbreviations: R1, first round; R2, second round; ST, Shashemene Town; SW, Shashemene Woreda.

^a^In ST, 270 residents received their first dose in R2; 40 183 received both doses (R1 + R2).

^b^In SW, 344 residents received their first dose in R2; 60 136 received both doses (R1 + R2).

**Table 4.** OCV Administrative Coverage Stratified by Kebele, Age Group, and Sex

Vaccination Site and Kebelle by Round	Target Population	No. by Age Group and Sex								Total No. Vaccinated	% of Coverage	1st Dose in R2	Both R1 and R2
		1–4 y		5–14 y		15–60 y		>60 y					
		M	F	M	F	M	F	M	F				
ST													
R1													
Abosto	10 500	1136	1242	1835	2109	1759	2699	32	29	10 841	103.2	n.a	n.a
Alelu	10 500	986	1078	1408	1613	1940	2847	425	415	10 712	102.0	n.a	n.a
Arada	12 250	1609	1598	2008	2171	2127	2454	200	153	12 320	100.6	n.a	n.a
Awasho	7000	795	886	1112	1326	1247	1722	40	55	7183	102.6	n.a	n.a
Total for R1	40 250	4526	4804	6363	7219	7073	9722	697	652	41 056	102.0	n.a	n.a
Sum per age group	n.a	9330		13 582		16 795		1349		n.a	n.a	n.a	n.a
Proportion of total (n = 40 250), %	n.a	23		34		42		3		n.a	n.a	n.a	n.a
R2													
Abosto	10 500	1099	1350	1726	1787	1809	2313	73	36	10 193	97.1	79	10 114
Alelu	10 500	1019	1367	1517	1502	1701	2391	681	541	10 719	102.1	10	10 709
Arada	12 250	1391	1667	2318	2466	1905	2470	116	91	12 424	101.4	148	12 276
Awasho	7000	908	871	1255	1426	1056	1511	46	44	7117	101.7	33	7084
Total for R2	40 250	4417	5255	6816	7181	6471	8685	916	712	40 453	100.5	270	40 183
Sum per age group	n.a	9672		13 997		15 156		1628		n.a	n.a	n.a	n.a
Proportion of total (n = 40 250), %	n.a	24		35		38		4		n.a	n.a	n.a	n.a
SW													
R1													
Abaro	10 785	1217	1278	2196	1882	1256	1380	140	33	9382	87.0	n.a	n.a
Alelu Ilu	8032	938	986	2048	1946	1312	1859	80	132	9301	115.8	n.a	n.a
Bulchana Danaba	6188	934	958	1557	1383	945	1082	56	70	6985	112.9	n.a	n.a
Bura Borama	6743	605	607	1045	1027	1214	1506	65	51	6120	90.8	n.a	n.a
Chabi Dida Gnata	6792	711	764	1206	1300	1154	1528	52	57	6772	99.7	n.a	n.a
Edola Burka	10 019	1217	1227	2081	2164	1291	2002	74	76	10 132	101.1	n.a	n.a
Faji Gole	9263	960	939	1380	1395	1748	1842	160	173	8597	92.8	n.a	n.a
Toga	3217	362	364	671	544	555	577	86	54	3213	99.9	n.a	n.a
Total for R1	61 039	6944	7123	12 184	11 641	9475	11 776	713	646	60 502	99.1	n.a	n.a
Sum per age group	n.a	14 067		23 825		21 251		1359		n.a	n.a	n.a	n.a
Proportion of total (n = 61 039), %	n.a	23		39		35		2		n.a	n.a	n.a	n.a
R2	n.a	n.a	n.a	n.a	n.a	n.a	n.a	n.a	n.a	n.a	n.a	n.a	n.a
Abaro	9382	1081	1147	2043	1962	1540	1417	118	65	9373	99.9	0	9373
Alelu Ilu	9301	1214	1374	1738	1955	1281	1462	147	168	9339	100.4	291	9048
Bulchana Danaba	6985	1102	1089	1360	1192	955	973	152	158	6981	99.9	0	6981
Bura Borama	6120	618	581	1134	1126	1140	1413	63	41	6116	99.9	0	6116
Chabi Dida Gnata	6772	711	762	1201	1301	1148	1525	53	57	6758	99.8	30	6728
Edola Burka	10 132	1219	1268	1846	2017	1502	2107	93	68	10 120	99.9	0	10 120
Faji Gole	8597	960	939	1380	1404	1740	1842	165	171	8601	100.0	0	8601
Toga	3213	332	402	708	651	489	428	116	66	3192	99.3	23	3169
Total for R1	60 502	7237	7562	11 410	11 608	9795	11 167	907	794	60 480	100.0	344	60 136
Sum per age group	n.a	14 799		23 018		20 962		1701		n.a	n.a	n.a	n.a
Proportion of total (n = 60 502), %	n.a	24		38		34		3		n.a	n.a	n.a	n.a

Unless otherwise specified, data represent no. of individuals by age group and sex.

Abbreviations: F, female; M, male; n.a, not applicable; R1, first round; R2, second round; y, years.

Of 40 250 people planned for vaccination in ST, populations aged 15–60 years had the highest proportions of OCV administration in both rounds (42% [16 795 people] in R1 and 38% [15 156] in R2), followed by adolescents aged 5–14 years (34% [13 582] and 35% [13 997], respectively), infants and younger children aged 1–4 years (23% [9330] and 24% [9672]), and lowest in older adults aged >60 years (3% [1349] and 4% [1628]) (Table 4). In SW, adolescents aged 5–14 years (39% [23 825 of 61 039] in R1 and 38% [23 018 of 60 502] in R2) had the highest administration of OCV, followed by older adolescents and adults aged 15–60 years (35% [21 251 of 61 039] and 34% [20 962 of 60 502], respectively), infants and younger children aged 1–4 years (23% [14 067 of 61 039] and 24% [14 799 of 60 502]), and older adults >60 years (2% [1359 of 61 039] and 3% [1701 of 60 502]).

### OCV Vaccination Coverage Estimate

A total of 112 and 165 households were surveyed in ST and SW, respectively (Table 5). The median number of household members (interquartile range) was 5.0 (4.0–6.0) in ST and 6 (4.0–7.0) in SW. In both ST and SW, about 53% of the household members were female: 52.96% (286 of 540) and 52.13% (477 of 915), respectively. In ST, 57.86% (313 of 541) of household members were aged ≥15 years, 29.94% (162 of 541) aged 5–14 years, and 12.20% (66 of 541) aged 1–4 years. In SW, 47.65% (436 of 915) were aged ≥15 years, 36.28% (332 of 915) aged 5–14 years, and 16.07% (147 of 915) aged 1–4 years age. In ST, 78% (95% CI: 73.06–82.94) of household members reported having received 2 doses of OCV, and 16.82% (95% CI: 12.36–21.28) reported not being vaccinated. About 51.57% (95% CI: 45.61–57.53) of household members reported single-dose OCV, but many also responded to 2-dose OCV; thus, differentiating individuals with only single-dose OCV was not straightforward. In SW, 83.06% (95% CI: 79.62–86.50) of household members reported having received 2-dose OCV, and 11.80% (95% CI: 8.84–14.76) reported not being vaccinated. Similar to findings in ST, the majority of respondents who reported single-dose OCV also responded to 2-dose OCV, so, it is not feasible to clearly differentiate those who received only single-dose OCV.

**Table 5.** OCV Coverage Survey: Characteristics of Participants and Coverage Estimate

Characteristic	Households Surveyed (N = 277)	
	ST (n = 112)	SW (n = 165)
**No. of household members, median (IQR)**	5.0 (4.0–6.0)	6.0 (4.0–7.0)
**Sex, no. (%)^a^**		
Female	286 (52.96)	477 (52.13)
Male	254 (47.04)	438 (47.87)
Total^b^	540	915
**Age group, no. (%)^a^**		
1–4 y	66 (12.20)	147 (16.07)
5–14 y	162 (29.94)	332 (36.28)
≥15 y	313 (57.86)	436 (47.65)
All ages^b^	541	915
**Estimated OCV coverage by age group, no. (% [95% CI])^a,c^**		
** All ages^c^**		
* * 2 doses	422 (78.00 [73.06–82.94])	760 (83.06 [79.62–86.50])
1 dose	279 (51.57 [45.61–57.53])	803 (87.76 [84.76–90.76])
0 dose^d^	91 (16.82 [12.36–21.28])	108 (11.80 [8.84–14.76])
** Age 1–4 y**		
2 doses	55 (83.33 [70.61–96.05])	115 (78.23 [68.80–87.66])
1 dose	38 (57.58 [40.72–74.44])	117 (79.59 [70.38–88.80])
0 dose^d^	10 (15.15 [2.92–27.38])	28 (19.05 [10.07–28.03])
**Age 5–14 y**		
2 doses	144 (88.89 [82.05–95.73])	302 (90.96 [86.60–95.32])
1 dose	81 (50.00 [39.11–60.89])	308 (92.77 [88.83–96.71])
0 dose^d^	11 (6.79 [1.31–12.27])	22 (5.05 [1.72–8.38])
**Age ≥15 y**		
2 doses	223 (71.25 [64.16–78.34])	343 (78.67 [73.23–84.11])
1 dose	160 (51.12 [43.29–58.95])	378 (86.70 [82.19–91.21])
* * 0 dose^d^	70 (22.36 [15.83–28.89])	58 (13.30 [8.79–17.81])

Abbreviations: CI, confidence interval; IQR, interquartile range; OCV, oral cholera vaccine; ST, Shashemene Town; SW, Shashemene Woreda; y, years.

^a^Analysis at individual level.

^b^Any discrepancies in total sums may be due to missing values in the data collected.

^c^Based on totals of 541 individuals for ST and 915 for SW.

^d^The “0 dose” results represent household members who were not vaccinated (n = 199).

Age group–stratified OCV coverage survey results showed the high coverage of 2-dose OCV across all age groups (Table 5). In ST, the 2-dose coverage rates were 83.33% (95% CI: 70.61–96.50) in 1–4-year-olds, 88.89% (95% CI: 82.05–95.73) in 5–14-year-olds, and 71.25% (95% CI: 64.16–78.34) in those aged ≥15 years. In SW, 2-dose coverage rates in the same 3 age groups were 78.23% (95% CI: 68.80–87.66), 90.96% (95% CI: 86.60–95.32), and 78.67% (95% CI: 73.23–84.11), respectively. Of children aged 1–4 years, about 15.15% (95% CI: 2.92–27.38) in ST and 19.05% (95% CI: 10.07–28.03) in SW were reported as not having received any OCV doses. Of household members aged 5–14 years, about 6.79% (95% CI: 1.31–12.27) in ST and 5.05% (95% CI: 1.72–8.38) in SW were reported as not vaccinated with OCV, compared to about 22.36% (95% CI: 15.83–28.89) and 13.30% (95% CI: 8.79–17.81) of those aged ≥15 years in ST and SW, respectively.

### Community Awareness and Acceptance of OCV Vaccination

In both ST and SW, health workers were the most influential players in community sensitization on the OCV vaccination campaign: 95.54% (107 of 112) of households in ST and 81.82% (135 of 165) in SW (Table 6). Next, community mobilizers (71.52% [118 of 165]), community leaders (55.76% [92 of 165]), and megaphones (44.24% [73 of 165]) were the key messengers in SW. In ST, health workers and megaphones (81.25% [91 of 112]) were predominant source of information, followed by community mobilization (35.71% [40 of 112]), community leaders (27.68% [31 of 112]), local government officers (14.29% [16 of 112]), radio (11.61% [13 of 112]), television (6.25% [7 of 112]), and religious leaders (1.79% [2 of 112]). In comparison, more diverse modes of communications were reportedly more effective for community sensitization in SW than in ST, such as local government officers (25.45% [42 of 165]), religious leaders (19.39% [32 of 165]), radio (17.58% [29 of 165]), and family members (12.12% [20 of 165]). On vaccine acceptance, the majority of households gave the reason for nonvaccination as nonavailability due to work during the vaccination period: 41.07% (46 of 112) in ST and 32.73% (54 of 165) in SW. Notably, 10.91% of SW households (18 of 165) reported that they were told not to be vaccinated, and 2.68% (3 of 112) and 3.64% (6 of 165) of households in ST and SW, respectively, gave fear of adverse events as the principal reason for not receiving OCV.

**Table 6.** Source of Information on OCV Vaccination Campaign and Principal Reasons for Not Receiving OCV

Sources and Reasons	Households Surveyed, No. (%)	
	ST (n = 112)	SW (n = 165)
**Source of information^a^**		
Health workers	107 (95.54)	135 (81.82)
Community leader	31 (27.68)	92 (55.76)
Religious leader	2 (1.79)	32 (19.39)
Local government officer	16 (14.29)	42 (25.45)
Community mobilizers	40 (35.71)	118 (71.52)
Family member	0 (0.00)	20 (12.12)
Megaphone	91 (81.25)	73 (44.24)
Radio	13 (11.61)	29 (17.58)
Television	7 (6.25)	1 (0.61)
Others	0 (0.00)	1 (0.61)
**Reason for nonvaccination^a^**		
Not available due to work during vaccination period	46 (41.07)	54 (32.73)
Ill during vaccination period	1 (0.89)	3 (1.82)
Did not know/hear about OCV vaccination	3 (2.68)	1 (0.61)
Vaccination post was not accessible	0 (0.00)	0 (0.00)
Vaccination post did not have OCV doses	0 (0.00)	0 (0.00)
Vaccination post did not have health workers	0 (0.00)	0 (0.00)
Was told not to receive vaccine	0 (0.00)	18 (10.91)
Heard that the vaccine is not safe for pregnant women	0 (0.00)	3 (1.82)
Fear of adverse event after vaccination	3 (2.68)	6 (3.64)
Other reasons	64 (57.14)	68 (41.21)

Abbreviations: OCV, oral cholera vaccine; ST, Shashemene Town; SW, Shashemene Woreda.

^a^Some respondents listed multiple sources of information or reasons for nonvaccination.

## DISCUSSION

OCV vaccination coverage was high. Overall administrative coverage in ST and SW showed that almost all populations in the vaccination target areas received OCV in both rounds. Some variations were noted by kebelle, such as Abaro, Bura Borama, and Faji Gole kebeles in SW, which had relatively lower administrative coverage rate in R1 of the vaccination campaign, though still nearly or above 90%. The mixed vaccination strategy, combining fixed posts and mobile teams, worked well. The daily vaccination spots varied depending on the vaccination flow. In the early morning, we initiated the vaccination at the planned fixed vaccination posts in the community. Later, we implemented mobile vaccination to reach target populations who could not come to the fixed posts. The mobile vaccination outreach spots were adjusted daily to reach the target populations.

The overall low dropout between doses may be attributable to the continued community sensitization before and during each round of vaccination campaign, as well as the daily monitoring of OCV administration compared with the microplan. Daily monitoring of OCV dose use and tracking of the number of people vaccinated versus the number planned in each planned kebelle were followed by subsequent action points to increase vaccine uptake, such as active outreach. Mobile teams went extra miles for active outreach, especially in remote rural villages of SW. The proportions of local residents who were vaccinated by age group reached >70% in adolescents and adults (aged 15–60 years) and children (aged 5–14 years), followed by infants and younger children (aged 1–4 years) and only 2–4% of those aged >60 years. This may generally correspond to the age structure of population in the background community.

Overall age group–stratified coverage survey revealed high OCV coverage across all age groups in both ST and SW. Older children and adolescents (aged 5–14 years) had the highest vaccine coverage at about 90% with 2 doses, and only 5–6% remained unvaccinated. Infants and younger children (aged 1–4 years) also showed high 2-dose OCV coverage at about 80%, though nonvaccination was reported for about 15% in ST and 19% in SW. For older adolescents and adults (aged ≥15 years), the 2-dose coverage ranged 71–79%, with 17% in ST and 12% in SW unvaccinated. These OCV coverage estimates show that when comparing ST and SW by age group rates, residents in SW rural area had higher 2-dose OCV coverage and lower nonvaccination (indicative of single-dose coverage) than the same age group in the ST urban area, except for the 1–4-year age group. Although the difference was not huge, it is worth noting these coverage survey findings when monitoring postvaccination cholera surveillance in these areas.

Health workers were critical in community sensitization on the OCV vaccination campaign in both urban and rural settings of Shashemene. This is highly attributable to the HEWs at the front line of Ethiopia's primary health system, who serve as a vital link between health sector and communities [28]. The Ethiopian government launched its Health Extension Program in 2004 to strengthen its engagement with community health workers, whereby 2 HEWs are assigned to each kebelle, the lowest administrative unit, with about 1000 households [29]. The HEWs, primarily supervised by the health center staff, are critical in community engagement in health-related education, including sensitization of local populations on any immunization plans, such as our mass OCV vaccination campaign. Furthermore, these HEWs work closely with the Women's Development Army (WDA) set-up in 2011, which extends the outreach of HEWs by grouping 5–6 neighboring households into teams, with each team selecting a WDA volunteer from a model household determined by healthy behavior adoption [29].

In the rural communities of Shashemene, community mobilizers, community leaders, and family members play a larger role. The main reason for nonvaccination was nonavailability during the vaccination campaign period in general, but this was not a big barrier as the vaccine coverage estimates were high. Notably, some rural communities were told not to be vaccinated, and small proportions of both urban and rural residents had fear of adverse events after vaccination. However, as cholera has been a public health concern in ST and SW over many years, the local communities in both ST and SW had fairly good awareness of cholera [30], which may also help explain the high OCV vaccination coverage in these areas.

Our study has a few limitations. First, the coverage survey was often based on the self-reported vaccination status during the household visits. Although the OCV vaccination cards were issued to individuals who were vaccinated at each round of the OCV vaccination campaign, these cards were often lost, damaged, or forgotten. Hence, coverage survey results may not capture the exact coverage rates. Nevertheless, as our vaccine coverage survey was conducted immediately after the vaccination campaign, the risk of recall bias may be low. Second, the coverage survey was answered by household heads for everyone in the household, which could not be verified if any responses were not accurate. Third, the coverage survey captured age group–stratified data for residents aged ≥15 years without further differentiating the older age group (aged >60 years), while the administrative coverage did show this breakdown. Thus, comparing administrative coverage and coverage survey results in the residents aged >60 years was not feasible.

Despite these limitations, our study demonstrated the successful roll-out of a preemptive 2-dose OCV vaccination campaign with high vaccination coverage in one of the cholera-endemic and high-priority hotspot areas in Ethiopia. In parallel, prospective sentinel healthcare facility–based cholera and diarrheal disease surveillance is underway in the surveillance catchment area that includes the OCV-vaccinated areas to evaluate the effectiveness and impact of this OCV vaccination. This could be also compared with any future studies on cholera vaccine schedules in Ethiopia, such as number of doses and dosing intervals, addressing the research needs highlighted in the Cholera Roadmap Research Agenda [31]. Monitoring and evaluation after vaccination, such as coverage surveys and impact assessment on disease burden, are important for future recommendations on OCV use.

## CONCLUSION

In conclusion, high OCV vaccination coverage was achieved in our preemptive 2-dose vaccination campaign. A mixed vaccination strategy including both fixed posts and mobile teams helped reach out to remote villages of the vaccination target areas. This, coupled with the daily monitoring of vaccination administrative coverage contributed to the high OCV coverage in both rounds of the campaign. Overall high community awareness on cholera may also have led to the high OCV uptake. Community sensitization was successful, and health workers were key players in promoting this OCV vaccination campaign, and their roles in other cholera prevention and response interventions could be critical to achieving public health goals. Vaccine acceptance and confidence in OCV was high, as exhibited in the high coverage rates, though a small portion of residents in urban and rural areas of Shashemene expressed fear of adverse event after immunization.

While overall awareness and perception toward cholera and OCV seems high in these communities, continued efforts in community engagement on the safety profile of OCV are recommended. Long-term multi-year cholera surveillance is needed to elucidate the impact and effectiveness of this vaccination. In further research, the impact and effectiveness of 2-dose OCV with a 2-week dose interval could be also compared with that of single-dose OCV or a delayed second dose OCV in Ethiopia, where reactive single-dose campaigns have been conducted in the recent years. A vaccine coverage survey is recommended for each vaccination campaigns. This, coupled with sustained and strengthened cholera surveillance to monitor the disease burden after vaccination, is essential for future decisions on OCV use.

## Supplementary Data

Supplementary materials are available at *Clinical Infectious Diseases* online. Consisting of data provided by the authors to benefit the reader, the posted materials are not copyedited and are the sole responsibility of the authors, so questions or comments should be addressed to the corresponding author.

## Supplementary Material

ciae233_Supplementary_Data

## Notes

***Acknowledgments.*** The authors acknowledge all the vaccination field team members and local government officials and healthcare professionals in Shashemene Town and Shashemene Woreda and Oromia Region Health Bureau for their active engagement and support of the OCV vaccination. We thank the local communities in the vaccination target areas for their sensitization and consent to take part in the vaccination coverage survey. We also extend appreciation to our research partners and dedicated staff at the ECCP study network in Ethiopia, including the EPSA for OCV dose storage in Addis Ababa and Hawassa under the cold chain management. Finally, thanks to Sujin Hwang for administrative support to the project, including procurement and delivery of OCV doses to Ethiopia, and to Bo-Sung Kim and Hae Kyung Cho for their operational support.

***Author contributions.*** S. E. P. and M. T. conceptualized the overall study design of the ECCP project. A. G., in discussion with ECCP team members and partners at Ethiopia Public Health Institute, put together the microplanning of OCV vaccination, under the supervision of S. E. P. and M. T. M. T., A. G., D. H., T. G., E. M. G., B. Y., O. D. M., Y. J., and S. E. P. (hereafter “supervisors”) and vaccination field teams in Shashemene Town and Shashemene Woreda participated in the OCV vaccination campaign launch, roll-out, and monitoring. The field teams recorded OCV vaccination registry, vaccination cards, and tally sheets for daily vaccination status documentation, supported by the supervisors. D. H. led the planning for the OCV vaccination coverage survey, with input from the ECCP team and review by S. E. P. and M. T. The supervisors and the research assistants participated in data collection for the vaccination coverage survey. All data collected were reviewed by the supervisors. D. M. and R. M. conducted the statistical work, and G. H. J. developed the REDCap system. S. E. P. and M. T. drafted and edited the manuscript, and all authors read and approved the final draft.

***Data sharing.*** All data relevant to the study are included in this article.

***Financial support*.** This study was supported by the Korea Support Committee for International Vaccine Institute (CHMTD05083-010), LG Electronics (CHMTD05083-020), and Community Chest of Korea (CHMTD05083-030). The International Vaccine Institute (IVI) acknowledges its donors, including the government of the Republic of Korea and the Swedish International Development Cooperation Agency.

***Supplement sponsorship.*** This article appears as part of the supplement “Ethiopia Cholera Control and Prevention (ECCP): Evidence-Generation Towards Global Roadmap to Ending Cholera,” sponsored by the IVI.
