# Supplementary material for: Coverage of Two-Dose Preemptive Cholera Mass Vaccination Campaign in High-Priority Hotspots in Shashemene, Oromia Region, Ethiopia
Source: Clin Infect Dis. 2024 Jul 12;79(Suppl 1):S33–42. doi: 10.1093/cid/ciae233 (PMC11244208; doi:10.1093/cid/ciae233)
Supplement: ciae233_Supplementary_Data [file ciae233_supplementary_data.docx]

**Supplementary Material 1. OCV Vaccination Card**


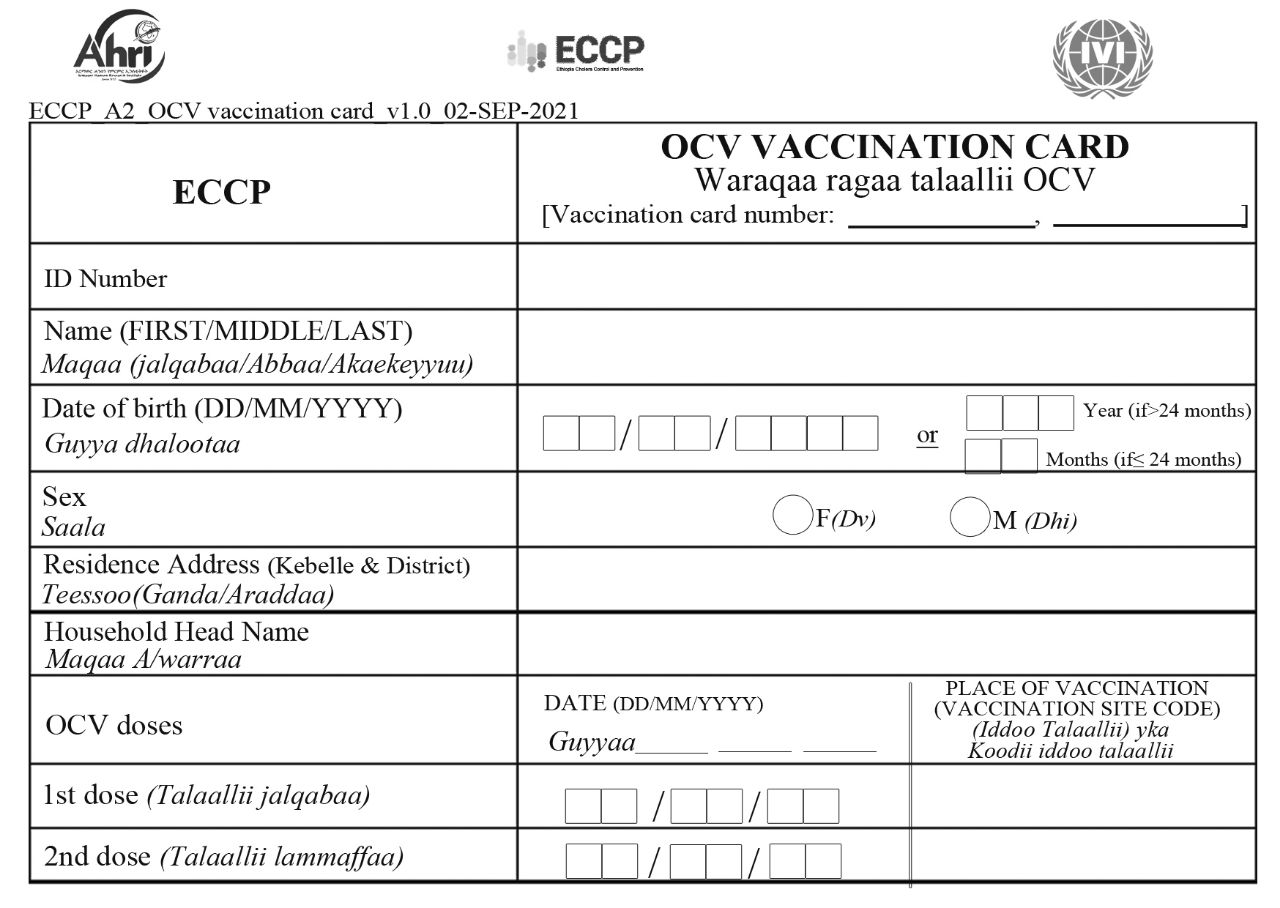


**Supplementary Material 2. OCV Vaccination Registry Logbook**

**
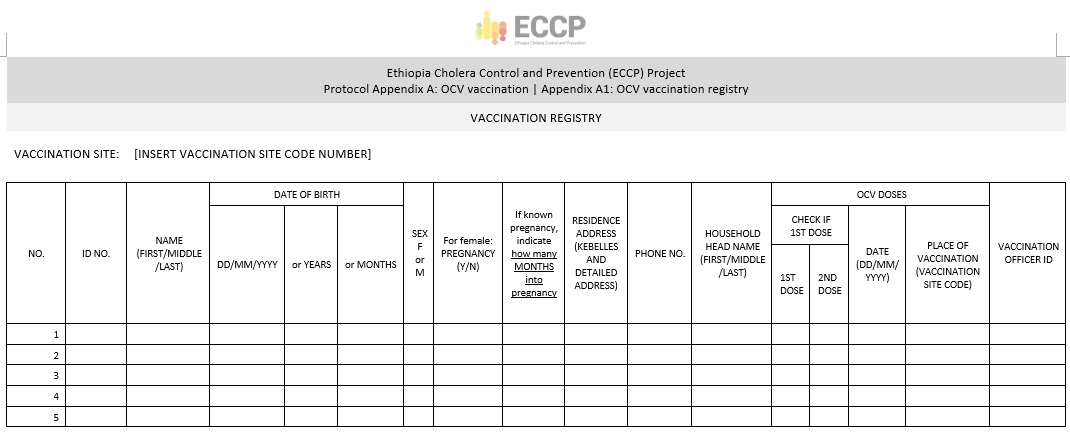
**

**Supplementary Material 3. OCV Vaccination Tally Sheet**


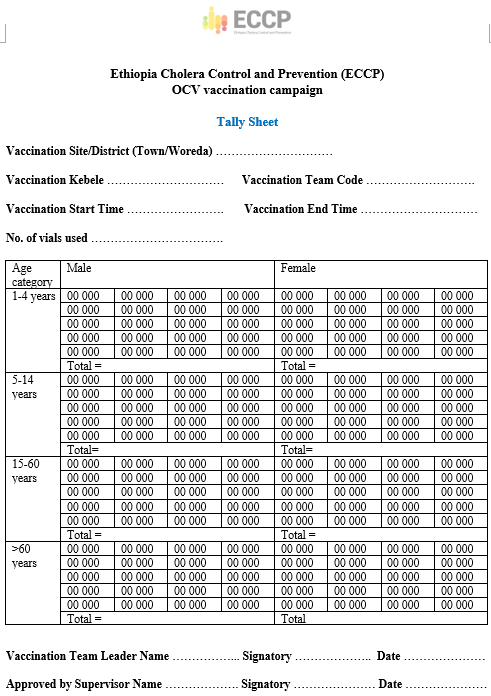


**Supplementary Material 4. OCV Vaccination Team Daily Reporting Format**


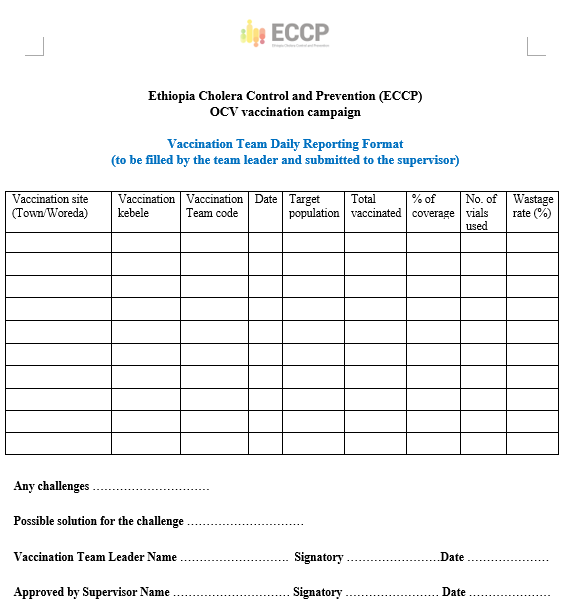


**Supplementary Material 5. ECCP OCV Vaccination Coverage Survey Questionnaire**


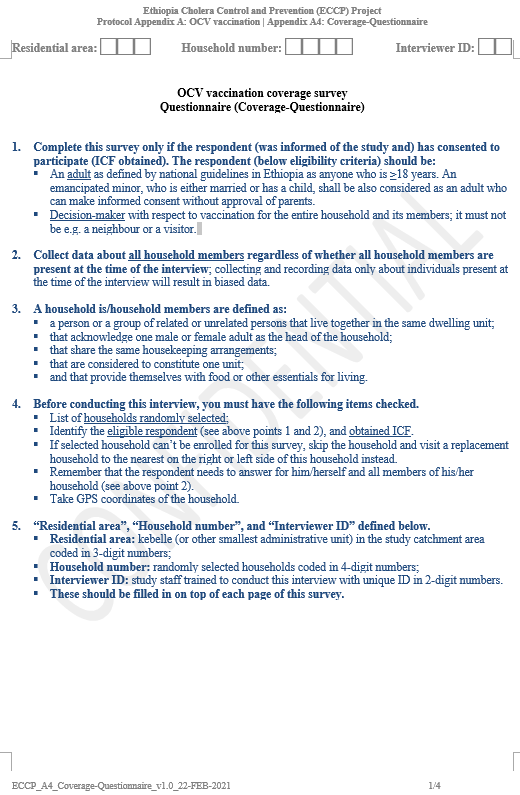


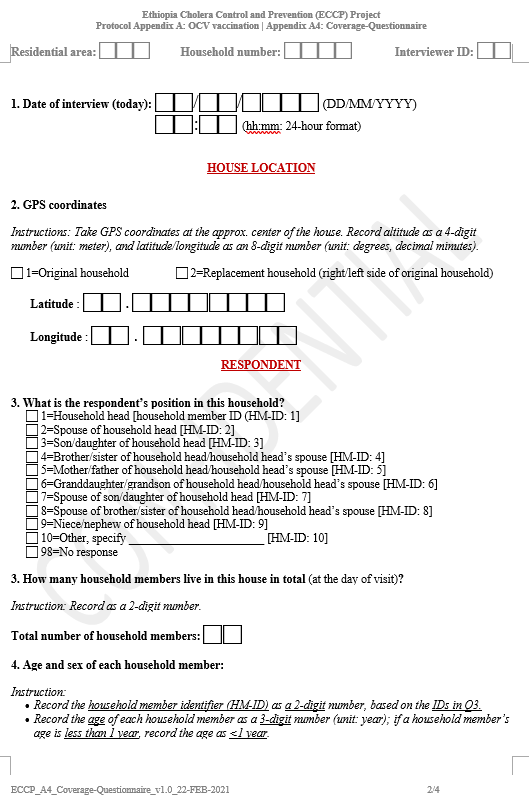


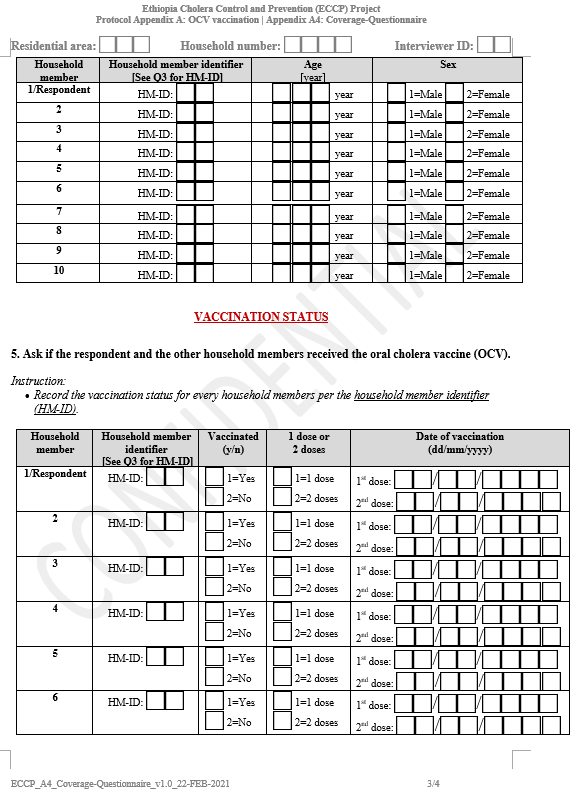


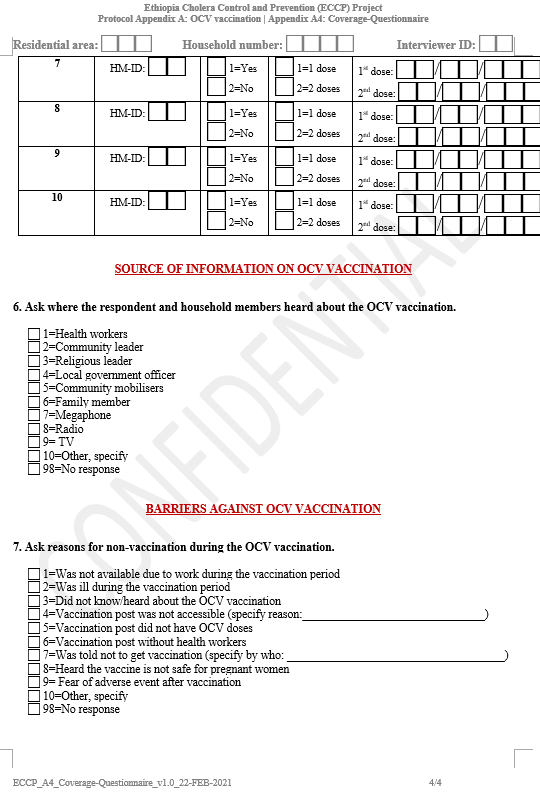


**Supplementary Table 1. OCV coverage survey households in Shashemene Town**

| **Kebele** | **Name of Ketena** | **No. of Population (2021)** | **No. of HHs^§^ in Ketena (2021)** | **No. of HHs for OCV coverage survey** |
| --- | --- | --- | --- | --- |
| **Abosto** |  |  |  |  |
|  | Demitu K6 | 7,836 | 637 | 16 |
|  | Demitu K10 | 3,051 | 248 | 6 |
|  | **TOTAL** | **10,887** | **885** | **22** |
| **Arada** |  |  |  |  |
|  | Demitu K3 | 7,847 | 665 | 16 |
|  | Kedija K1&2 | 4,532 | 384 | 9 |
|  | **TOTAL** | **12,379** | **1,049** | **25** |
|  |  |  |  |  |
| **Awasho** |  |  |  |  |
|  | Mekiya | **7,630** | **700** | **18** |
|  |  |  |  |  |
| **Alelu** |  |  |  |  |
|  | Arabe | 3,809 | 577 | 14 |
|  | Masho K6 | 4,013 | 608 | 15 |
|  | Sr Shage Hussen | 2,033 | 308 | 8 |
|  | Sr Shage | 2,759 | 418 | 10 |
|  | **TOTAL** | **12,614** | **1,911** | **47** |
|  |  |  |  |  |
|  | **GRAND TOTAL** | **43,510** | **4,545** | **112** |

Footnote: ^§^ HHs: households

**Supplementary Table 2. OCV coverage survey households in Shashemene Woreda**

| **Cluster** | **Name of Kebele** | **No. of Population (2021)** | **No. of HHs**^§^ **in kebele (2021)** | **No. of HHs for OCV coverage survey** | |
| --- | --- | --- | --- | --- | --- |
| **Faji Gole PHCU*** | | | | |  |
|  | Faji Gole | 9,263 | 1,994 | 25 | |
|  | Allelu Illu | 8,032 | 1,729 | 22 | |
|  | **TOTAL** | **17,295** | **3,723** | **47** | |
| **Harabate PHCU** | | | | |  |
|  | E/Burka | 10,019 | 2,157 | 27 | |
|  | Abaro | 10,785 | 2,322 | 29 | |
|  | **TOTAL** | **2,084** | **1,049** | **56** | |
|  |  |  |  |  | |
| **Toga PHCU** | | | | |  |
|  | B/Danaba | 6,188 | 1,088 | 14 | |
|  | Toga | 3,217 | 693 | 9 | |
|  | **TOTAL** | **9,405** | **1,781** | **23** | |
| **Chabi PHCU** | | | | |  |
|  | Chabi Dida Gnata | 6,792 | 1,462 | 18 | |
|  | Bura Borema | 6,743 | 1,451 | 18 | |
|  | **TOTAL** | **13,535** | **2,913** | **36** | |
|  | **GRAND TOTAL** | **61,039** | **12,896** | **162** | |

Footnote: ^§^ HHs: Households. * PHCU: Primary Health Care Unit
